# Applying a web-based self-help intervention for bulimia nervosa in routine care: Study protocol for a randomized controlled trial

**DOI:** 10.1016/j.invent.2022.100512

**Published:** 2022-02-17

**Authors:** Steffen Hartmann, Luise Pruessner, Julian A. Rubel, Christopher Lalk, Sven Barnow, Christina Timm

**Affiliations:** a Department of Psychology, Heidelberg University, Hauptstr. 47-51, 69117 Heidelberg, Germany; b Psychotherapy Research Unit, University of Giessen, Otto-Behaghel-Straße 10, 35394 Giessen, Germany

**Keywords:** Bulimia nervosa, Web-based interventions, Randomized controlled trial, Emotion regulation, Ecological momentary assessment, Internet-based self-help

## Abstract

**Background:**

Individuals with bulimia nervosa (BN) experience persistent episodes of binge eating and inappropriate compensatory behavior associated with impaired physical and mental health. Despite the existence of effective treatments, many individuals with BN remain untreated, leading to a high burden and an increased risk of chronicity. Web-based interventions may help facilitate access to evidence-based treatments for BN by reducing barriers to the health care system.

**Methods:**

The present study will investigate the effectiveness of a web-based self-help intervention for BN in a two-armed randomized controlled trial. Individuals diagnosed with BN (*N* = 152) will be randomly assigned to either (1) an intervention group receiving a 12-week web-based intervention or (2) a waitlist control group with delayed access to the intervention. Further assessments will be scheduled 6 (mid-treatment) and 12 (post-treatment) weeks after baseline. Changes in the number of binge eating episodes and compensatory behaviors will be examined as primary outcomes. Secondary outcomes include global eating pathology, functional impairments, well-being, comorbid psychopathology, self-esteem, and emotion regulation abilities.

**Discussion:**

Adding web-based interventions into routine care is a promising approach to overcome the existing treatment gap for patients with BN. Therefore, the current study will test the effectiveness of a web-based intervention for BN under standard clinical care settings.

**Trial registration:**

*ClinicalTrials.gov*, Identifier: NCT04876196 (registered on May 6th, 2021).

## Highlights

• Although bulimia nervosa is associated with marked impairments, there is a treatment gap for patients in routine care.
• Integrating web-based interventions into routine care might facilitate access to treatment for patients with bulimia nervosa.
• A web-based self-help intervention will be evaluated by examining changes in core symptoms of bulimia nervosa.
• Secondary outcomes include measures of psychopthology and psychosocial functioning as well as potential mechanisms of change.
• Weekly questionnaires and ecological momentary assessment will increase the external validity and temporal resolution of results.

## 1 Introduction

### 1.1 Background and rationale

Bulimia nervosa (BN) is an eating disorder with an estimated lifetime prevalence of about 0.8% (Kessler et al., 2013). Core symptoms of BN include recurrent binge eating episodes, inappropriate compensatory behaviors and maladaptive over-evaluations of body shape and weight (APA, 2013; Forrest et al., 2018). These symptoms lead to a high psychological burden as well as marked impairments in physical health, social integration, professional performance, and overall quality of life (Erskine et al., 2016; Stice et al., 2009). As BN is associated with high societal costs, increased mortality, and often a chronic course (APA, 2013; Arcelus et al., 2011; Bode et al., 2017; Nagl et al., 2016), there is a high need to identify effective treatments (Kazdin et al., 2017).

Previous research provides evidence for the effectiveness of cognitive-behavioral therapy (CBT) for BN (Linardon et al., 2017b; Svaldi et al., 2019), and CBT is the treatment of choice according to national European treatment guidelines (Herpertz and Herpertz-Dahlmann, 2017; NICE, 2017). Nonetheless, despite the existence of effective treatments for BN such as CBT, most patients remain untreated or do not receive evidence-based therapies, increasing the risk for chronicity and the burden of illness (Grange and Loeb, 2007; Mack et al., 2014; Striegel Weissman and Rosselli, 2017).

Concerning the causes of this treatment gap, previous research indicates that a combination of patient-associated and healthcare-related barriers hinders the help-seeking process (Ali et al., 2016; Erskine et al., 2016; Grange and Loeb, 2007; Striegel Weissman and Rosselli, 2017). On the patient's side, limiting factors include fear of stigmatization or change, feelings of shame and guilt, poor motivation, or a low mental health literacy (Ali et al., 2016; Becker et al., 2010; Grange and Loeb, 2007; Mond, 2014; Puhl and Suh, 2015; Regan et al., 2017). Additional barriers of the health care system lead to long waiting periods and many patients receiving unspecific care (Hart et al., 2011; Kazdin et al., 2017; Mack et al., 2014). While low treatment rates are common for many mental disorders, eating disorders are even less likely to be adequately treated (Andrade et al., 2014; Kessler et al., 2013), and it often takes years from the occurrence of the first symptoms to treatment uptake (Andrade et al., 2014; Hamilton et al., 2021; Puhl and Suh, 2015; Roehrig and McLean, 2010). Consequently, targeting treatment barriers is fundamental to improving routine care for BN.

Web-based self-help interventions might help facilitate access to mental health care by allowing higher temporal flexibility, being location-independent and cost-efficient while still providing the possibility of individual treatment adaptations (Andersson and Titov, 2014; Beintner et al., 2014; Pittock et al., 2018; Wagner et al., 2015). Besides, these interventions have the potential to enhance the routine care for BN, since they can bridge waiting periods for face-to-face psychotherapy, advance the transition from inpatient to outpatient care, or provide a low-threshold alternative to previous therapy offers (Aardoom et al., 2013; Dölemeyer et al., 2013; Vollert et al., 2019).

Regarding their effectiveness, first studies suggest that web-based self-help interventions can successfully reduce BN symptoms (Haderlein, 2022; Linardon et al., 2020; Pittock et al., 2018; Taylor et al., 2021). Still, most research on web-based interventions focused on other mental disorders (Josephine et al., 2017; Richards et al., 2015) and current systematic reviews respectively meta-analyses conclude that more research on the effectiveness of web-based treatments for patients with BN is needed (Beintner et al., 2014; Haderlein, 2022; Linardon et al., 2020; Pittock et al., 2018; Taylor et al., 2021). As such, previous RCT studies examined specific interventions such as group or dissonance-based therapies (e.g., Green et al., 2018; Zerwas et al., 2017), were restricted to a subgroup of patients (e.g., Sanchez-Ortiz et al., 2011; Wagner et al., 2013) or included a mixture of sub- and full-threshold BN patients (e.g., Ruwaard et al., 2013). Consequently, the generalizability of these results is limited and despite the named benefits of integrating web-based CBT interventions into routine care, studies on their effectiveness in this setting are missing.

The present randomized controlled trial (RCT) addresses this research gap by evaluating whether applying a 12-week web-based self-help intervention in routine care will lead to a decline in eating disorder symptoms and improvements in the quality of life in patients with BN compared to a waitlist control group. Consequently, the primary goal of the current trial is to expand previous findings to a more representative sample. As such, we aim at overcoming the limitations of earlier studies by investigating the effectiveness of a web-based self-help program in a sample of full-threshold BN patients that would typically receive unspecialized, non-evidence-based care or no treatment at all (Hay et al., 2020; Kessler et al., 2013; Mack et al., 2014).

Our second goal is to explore how web-based interventions can affect symptom changes and whether patient characteristics influence treatment outcomes. So far, knowledge on moderators, mediators, and predictors of treatment success for internet-based CBT is still limited (Linardon et al., 2017a; Taylor et al., 2021). Thus, we aim at gaining a better understanding of how and why web-based interventions can reduce eating disorder symptoms by investigating possible mechanisms of change. Theoretical models, such as the transdiagnostic theory of eating disorders, and empirical findings stress the importance of psychopathological processes in the development and maintenance of eating disorders (Fairburn et al., 2003; Linardon et al., 2019; MacDonald and Trottier, 2019; Svaldi et al., 2019). Therefore, we will test self-esteem, emotion regulation, and comorbid psychopathology as possible mechanisms of change to address this research gap and help to improve internet-based interventions in the future.

Finally, our trial will incorporate weekly symptom measurements and ecological momentary assessment (EMA) to increase the validity of results and obtain a more nuanced understanding of treatment effects (Munsch et al., 2009; Pruessner et al., 2022; Shiffman et al., 2008).

## 2 Methods

### 2.1 Objectives and hypotheses

This study will investigate the effectiveness of a 12-week self-help program, *Selfapy* for BN, which is based on methods of CBT and was designed to directly target BN symptoms as well as topics related to BN such as emotion regulation, stress management, and self-esteem (Knaevelsrud et al., 2016; Sipos and Schweiger, 2016; Tuschen-Caffier and Florin, 2012; Vocks et al., 2005; Waadt et al., 2013). We expect that participants receiving the treatment will show a substantially greater decline in the number of binge eating episodes and the frequency of compensatory behaviors over the 12 weeks of treatment compared to a waitlist control condition. Additionally, we presume that the intervention will be associated with a higher reduction in global eating disorder symptoms. Considering changes in the quality of life, we will further expect a reduction in psychosocial impairments, a higher increase in well-being, and better restoration of work capacity (Agh et al., 2016). Finally, to better understand possible mechanisms of change, we will explore whether the web-based intervention for BN will lead to a greater decline in comorbid psychopathology and more substantial improvements in global self-esteem as well as the ability to regulate emotions after the treatment (Fairburn et al., 2003; Linardon et al., 2019; MacDonald and Trottier, 2019; Svaldi et al., 2019).

The present trial is part of a research project at Heidelberg University evaluating the effectiveness of two different web-based self-help interventions for eating disorders. While the current trial examines a self-help program for BN, a parallel trial will focus on treating binge eating disorders (Pruessner et al., 2022). The different interventions were developed to target the core DSM-5 symptomatology of these diagnoses. Therefore, the training for BN addresses recurrent compensatory behaviors and maladaptive self-evaluations besides binge eating episodes. As these symptoms are not characteristic of binge eating disorder, they are not included in the web-based binge eating disorder intervention. The two studies will be conducted simultaneously to optimize the study administration procedure, leading to highly similar trial designs. However, considering the different contents of the intervention and meta-analytic findings showing different effect sizes of psychological treatments for BN and binge eating disorders (Beintner et al., 2014; Castellini et al., 2012), two separate trials will be conducted.

### 2.2 Participants and recruitment

The recruitment will include online and offline advertisements to reach a high number of German-speaking individuals potentially eligible for our study. Online advertisements will include postings on the intervention provider's website (https://www.selfapy.de) and social media. Further study information will be shared via mailing lists, and a waitlist of subjects interested in the intervention will be contacted. Moreover, information material on paper will be distributed in Germany.

Interested individuals will be referred to a webpage containing a detailed study description and the possibility to register for the trial. On this page, all participants are asked to provide informed consent after receiving details about the study procedure and the possible 12-week waiting period for the intervention. The information concerning the waiting time is repeated during the baseline interview and assessment, so participants can ask questions about it. After a short screening questionnaire, interested individuals can make an appointment for the clinical interview.

Eligible participants included in the study will receive a financial reimbursement of 30€ upon completing all study assessments (baseline, mid-treatment, post-treatment). The patient characteristics based on the PICO framework (Schardt et al., 2007) can be found in Table S1 of our supplementary materials. The recruitment for the present trial will be combined with the binge eating disorder study conducted at Heidelberg University (Pruessner et al., 2022).

#### 2.2.1 Eligibility criteria

Eligible participants will need to (1) be aged from 18 to 65, (2) have adequate German-language skills (C1), (3) have a smartphone with permanent internet access during the study period, and (4) be diagnosed with BN according to the Diagnostic and Statistical Manual of Mental Disorders (DSM-5) criteria during the clinical interview (APA, 2013). Exclusion criteria are (1) a Body Mass Index (BMI) below 18.5, (2) currently receiving psychotherapy or pharmacotherapy for eating disorders at baseline, (3) a comorbid bipolar disorder or psychotic disorder, (4) an acute substance dependence, (5) a current severe depressive episode, and (6) acute suicidality. As web-based self-help interventions are not indicated for patients with severe psychiatric disorders, these comorbidities were selected as exclusion criteria (von Brachel et al., 2014). Individuals excluded after the clinical interview will be encouraged to seek professional help and receive a document containing potential alternative treatments via email. When individuals fulfill the criteria for binge eating disorder according to DSM-5, they will be referred to our parallel study evaluating a web-based intervention for this disorder (Pruessner et al., 2022). To represent routine care, patients diagnosed with BN but primarily fulfilling the criteria for other mental disorders will also be included in the study. Excluding individuals currently receiving psychotherapy or pharmacological treatment for eating disorders will prevent systematic pre-treatment group differences regarding healthcare services utilization and thus will allow us to attribute differences in outcome changes to the intervention. Due to ethical reasons and in order to maximize our external validity, all participants will be allowed to begin other treatments, including pharmacological and psychological therapies, after randomization. These additional treatments will be assessed throughout the trial.

### 2.3 Trial design

**Fig. 1:**
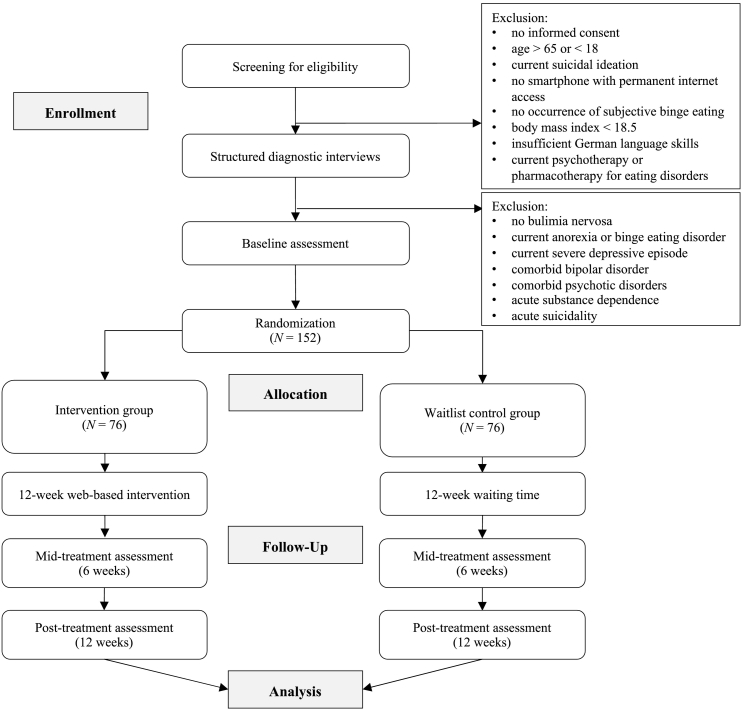
CONSORT flow of participants randomly assigned to a web-based intervention for bulimia nervosa or a waitlist control condition.

A two-armed, randomized controlled trial with a waiting control group will be conducted to investigate the effectiveness of the web-based intervention *Selfapy* for BN. A CONSORT flow diagram (Altman et al., 2001) for the study is presented in Fig. 1. Eligible subjects based on the structured diagnostic interview will be randomly assigned either to (1) an intervention group (IG) including the immediate access to the online self-help intervention for BN or (2) a waitlist control group (CG) with delayed access to the intervention (12 weeks).

#### 2.3.1 Randomization and blinding

Each participant completing the baseline assessment will be randomized by an independent researcher not involved in the project using a computer-based algorithm provided by the online platform *SoSci Survey* (Leiner, 2021) with a 1:1 ratio. Participants will be automatically informed about the randomization result. Therefore, interviews will be conducted blindly, as the clinical interviewers do not know to which group the interviewed participant will be allocated (allocation sequence concealment; Altman and Schulz, 2001). Participants are not explicitly informed that there is an intervention and a control group; instead, they are told that the waiting period for the intervention randomly varies between 0 and 12 weeks. After randomization, each participant will immediately receive an email either including the access code for the intervention (IG) or the information that the intervention will start in 12 weeks (CG).

Group allocation will be masked during the data analyses to minimize possible bias concerning the statistical procedure. For this purpose, an independent researcher will delete all information in the data set indicating the group allocation. Therefore, the evaluator does not know which expression of the group variable represents the IG or the CG.

#### 2.3.2 Intervention

The web-based intervention *Selfapy* for BN is a 12-week self-help program employing evidence-based methods and exercises derived from CBT (Knaevelsrud et al., 2016; Sipos and Schweiger, 2016; Tuschen-Caffier and Florin, 2012; Vocks et al., 2005; Waadt et al., 2013). Based on a general diathesis-stress model, the intervention aims at improving the participants' understanding of risk factors and their coping abilities. After introducing this model, different topics such as *eating behavior*, *maladaptive cognitions*, *emotion regulation*, and *stress management* are addressed in each module. Core exercises include eating protocols, behavioral analysis, cognitive restructuring, and exercises derived from stress-management trainings and mindfulness-based interventions, such as body scans (Schug, 2016). The exercises consist of informative texts, audio files, videos, and interactive elements, for instance, open text boxes. Addressing the diathesis-stress model according to which individual combinations of stressors and risk factors lead to the development of a mental disorder, the course is individually adaptable. As such, the intervention is split into a core course with six mandatory modules and an optional part with six individually selectable specialization areas. Table 1 gives an overview of the core course, the optional areas of specialization and the suggested treatment course.

**Table 1:** Content of the 12-week web-based intervention (Selfapy) for bulimia nervosa.

Module	Exercises
**Core course**	
1. *Goal-Setting*	Describing eating behavior and setting personal goals concerning the intervention
2. *Psychoeducation*	Psychoeducation, recognizing triggers and causes of bulimic behavior, eating protocols
3. *Eating Behavior*	Strategies to prevent bulimic behaviors, short- and long-term consequences of bulimic behaviors
4. *Negative Thoughts*	Identifying and replacing automatic negative thoughts associated with bulimic behavior, falsifying negative cognitions
5. *Emotion Regulation*	Regulating negative emotions to prevent bulimic behavior, identifying emotions, training regulatory strategies
6. *Stress Management*	Improving stress management, defusing stress-increasing thoughts, relaxation (e.g., Progressive Muscle Relaxation)
**Optional Content**	
7. *Self-Esteem*	Training self-confidence and self-acceptance to effectively deal with everyday problems
8. *Resources*	Recognizing personal resources and strengths, discovering new sources of resilience, increasing positive activities
9. *Social Environment*	Optimizing social support and strengthening social competencies
10. *Mindfulness*	Formal and informal mindfulness exercises, integrating mindfulness into everyday life
11. *Problem Solving*	Training problem-solving strategies for challenging situations in daily life
12. *Relapse Prevention*	Relapse prevention strategies, goals for further practice, “relapse bag” to avoid future bulimic behavior

Participants can access the *Selfapy* platform online via desktop or a mobile browser (Linardon et al., 2021). After registration, all modules are freely available to the users for 12 months. However, the program suggests following the course in the intended order within 12 weeks. Participants work on the course contents individually and can use an integrated messenger function to ask questions concerning the exercises or receive technical support. Additionally, a psychologist tracks the participant's progress and monitors undesirable developments, such as suicidal tendencies. Participants not engaging with the intervention after receiving the access code, will be reminded to start the program via e-mail.

Regarding data security, the provider underlies the General Data Protection Regulation of the European Union, and all data will be encrypted via Transport Layer Security and securely saved on servers in Germany. Participants can only access the intervention with their passwords. The intervention provider conducted several usability testing phases before the study started, and the intervention was evaluated during a pilot phase incorporating the feedback of patients with BN.

#### 2.3.3 Control group

Individuals allocated to the control group will not have access to the web-based intervention during the 12-week study period. After finishing the last assessment, they will automatically receive an email with the access code to the intervention. Participants in both groups are allowed to receive any other treatment during the study period, including pharmacological and psychological treatments. These additional treatments will be measured at each assessment.

### 2.4 Measures

#### 2.4.1 Eligibility screening

During an initial eligibility screening, individuals are asked to report their age, weight, height, permanent internet access, current psychotherapy or pharmacotherapy for eating disorders, the occurrence of core eating disorder behaviors (using the *Patient Health Questionnaire*; Löwe et al., 2002) and the risk for suicidality (using the *Ask Suicide*-*Screening Questions*; Horowitz et al., 2020). Non-eligible persons will be informed that they do not fulfill the inclusion criteria and are invited to participate in other studies. When a risk of suicidality is indicated, participants will be referred to other health care services.

#### 2.4.2 Structured clinical interviews

For further testing of the inclusion and exclusion criteria, structured clinical interviews will be conducted by an independent and trained researcher from Heidelberg University via a video-call platform or telephone. The interview will consist of two parts, first, the *Eating Disorders Examination Interview* (EDE; Hilbert et al., 2004) will be administered to assess the DSM-5 criteria for BN and exclude possible diagnoses of binge eating disorders or anorexia nervosa. For determining whether the DSM-5 criteria for exclusionary comorbid diagnoses, i.e., severe depressive episodes, bipolar disorders, substance use disorders, acute suicidality, and psychotic disorders, are met, we will use the *Diagnostic Interview for Psychological Disorders-Open Access* (DIPS-OA, Margraf et al., 2017). Each interview will be supervised by a licensed psychotherapist and discussed within the research team to ensure high diagnostic quality. Additionally, it is planned to calculate interrater reliability by independently coding 20 interviews by two different research team members.

#### 2.4.3 Outcomes

Primary and secondary outcome measures will be assessed at baseline (study entrance), six weeks after baseline (mid-treatment), and 12 weeks after baseline (post-treatment). Moreover, eating behavior, binge eating episodes, and compensatory behavior will be monitored weekly to assess changes in symptoms with a higher resolution. EMA will be scheduled pre- and post-treatment to measure eating disorder symptoms in daily life. All measures will be collected online using the assessment platform *SoSci Survey* (Leiner, 2021) and made available to participants at http://www.s2survey.net/. An overview of the clinical outcome measures over time based on the SPIRIT guidelines (Chan et al., 2013) is provided in Table 2.

**Table 2:** SPIRIT schedule of the randomized controlled trial

	Study period				
	Enrollment	Allocation	Post-allocation		Close-out
		*Study entrance*	*Weekly*	*Mid-treatment*	*Post-treatment*
Timepoint	*−t*_*1*_	*t*_*1*_	*t*_*1*_–*t*_*12*_	*t*_*2*_	*t*_*3*_
**Enrollment:**					
Eligibility screen	*+*				
Informed consent	*+*				
**Clinical interviews**					
Eating Disorder Examination Interview (EDE)	*+*				
Diagnostic Interview for Psychological Disorders (DIPS-OA)	*+*				
**Allocation**		*+*			
**INTERVENTION:**					
Web-based intervention *Selfapy* for bulimia nervosa			*+*	*+*	
Waiting time			*+*	*+*	
**ASSESSMENTS:**					
**Primary confirmatory outcome**					
Number of Binge Eating and Compensatory Behavior Episodes (EDE-Q)		*+*		*+*	*+*
**Secondary confirmatory outcomes**					
Eating Disorder Examination Questionnaire (EDE-Q)		*+*		*+*	*+*
Weekly Occurrence of Binge Eating Episodes (WBQ)		*+*	*+*	*+*	*+*
Clinical Impairment Assessment (CIA)		*+*		*+*	*+*
*i*MTA Productivity Cost Questionnaire (*i*PCQ)		*+*		*+*	*+*
World Health Organization Well-Being Index (WHO-5)		*+*		*+*	*+*
**Secondary exploratory outcomes**					
Patient Health Questionnaire Depression Scale (PHQ-9)		*+*		*+*	*+*
Generalized Anxiety Disorder Scale (GAD-7)		*+*		*+*	*+*
Difficulties in Emotion Regulation Scale (DERS)		*+*		*+*	*+*
Rosenberg Self-Esteem Scale (RSES)		*+*		*+*	*+*
Heidelberg Form for Emotion Regulation Strategies (HFERST)		*+*		*+*	*+*
Ecological Momentary Assessment (EMA)		*+*			*+*
**Other measures**					
Client Sociodemographic Service Receipt Inventory (CSSRI)		*+*		*+*	*+*
Negative Effects Questionnaire (NEQ)				*+*	*+*
Attitudes Towards Online Interventions (APOI)		*+*			*+*
Patients' Therapy Expectation and Evaluation Scale (PATHEV)		*+*		*+*	*+*

#### 2.4.4 Primary confirmatory outcomes

• Changes in the frequency of bulimic behaviors within the last 28 days: Based on previous meta-analyses and studies with a similar study design, the changes in the frequency of binge-eating episodes and compensatory behavior over the preceding four weeks were selected as the primary outcomes (Svaldi et al., 2019; Linardon et al., 2020; Wagner et al., 2013; Ruwaard et al., 2013). The *Eating Disorders Examination Questionnaire* (EDE-Q; Hilbert et al., 2007) will be used to assess the number of binge eating episodes and compensatory behaviors within the last 28 days based on the DSM-5 (APA, 2013). The EDE-Q has shown acceptable test-retest reliability (r > .71) regarding the assessment of bulimic behaviors (Rø et al., 2010).

#### 2.4.5 Secondary confirmatory outcomes

• Changes in global eating psychopathology: Reductions in global eating psychopathology will be investigated using the total score of the EDE-Q (Hilbert et al., 2007). The EDE-Q includes 22 items on four subscales that reliably capture the dimensions of weight concern, shape concern, eating concern, and restraint on a 7-point Likert scale (subscales: .85 ≤ Cronbach's α ≤ .93; overall α = .97; Hilbert et al., 2007).
• Changes in the weekly frequency of bulimic behavior and regular eating: The *Weekly Binges Questionnaire* (WBQ; Munsch et al., 2007; Munsch et al., 2019) assesses the frequency of binge eating episodes, compensatory behavior, and regular eating by asking participants to count the number of binge eating episodes, the number of compensatory behaviors, and the number of days with regular eating habits during the last week. Thus, the weekly questionnaires throughout the study period will allow us to monitor the occurrence and frequency of bulimic behaviors and assess changes with a higher temporal resolution. Reminders for the WBQ will be sent via text messages.
• Changes in eating-disorder-related daily difficulties: To assess reductions in clinical impairments specific to eating disorders, we will employ the *Clinical Impairment Assessment* scale (CIA; Bohn et al., 2008), which measures overall and domain-specific eating-disorder related impairments (i.e., cognitive, social, and personal). The CIA contains 16 items on a 7-point Likert scale and demonstrated excellent internal consistency (Cronbach's α = .97), construct validity, and sensitivity to change (Bohn et al., 2008).
• Changes in well-being: The *World Health Organization-Five Questionnaire* (WHO-5; Bech et al., 2003) will be used to assess changes in general well-being. The WHO-5 consists of five items rated on a 6-point scale and has an excellent internal consistency of Cronbach's α = .92 (Brähler et al., 2007).
• Changes in work capacity: In line with the increasing significance of health economic evaluations (Jenkins et al., 2021; Safi et al., 2022; Watson et al., 2018), improvements in work capacity and productivity will be measured based on the *iMTA Productivity Cost Questionnaire* (iPCQ; Bouwmans et al., 2013; Bouwmans et al., 2015). The iPCQ examines long-term (>2 weeks) and short-term (<2 weeks) absences from work and productivity losses as a result of sickness-related restrictions in work efficiency. Test-retest reliability of the iPCQ is excellent concerning the reported number of sick leave days (ICC = .83) and moderate regarding the number of days at work while impeded (ICC = .56) and efficiency rates (ICC = .73; Bouwmans et al., 2013).

#### 2.4.6 Secondary exploratory outcomes

• Changes in comorbid depressive symptoms: To capture a possible reduction in comorbid depressive symptoms, we will employ the depression module of the *Patient Health Questionnaire-9* (PHQ-9; Kroenke et al., 2001). The PHQ-9 is a valid self-administered questionnaire that captures the severity of depressive symptoms on nine 4-point Likert-scaled items based on the DSM-IV criteria and has an internal consistency of Cronbach's α = .86 (Kroenke et al., 2001).
• Changes in comorbid anxiety symptoms: Comorbid anxiety symptoms will be measured with the *General Anxiety Disorder Scale* (GAD-7; Löwe et al., 2008). The GAD-7 consists of seven items answered on a 4-point scale and is a valid and reliable questionnaire for assessing generalized anxiety disorder symptoms (Cronbach's α = .89).
• Changes in self-esteem: Self-esteem improvements will be analyzed as essential treatment target in patients with eating disorders (Linardon et al., 2019). For this purpose, the *Rosenberg Self-Esteem Scale* will be used (RSES; Roth et al., 2008), consisting of 10 items answered on a 4-point scale and showing good internal consistency (α = .88; Roth et al., 2008).
• Changes in emotion regulation difficulties: Possible decreases in emotion regulation difficulties following the intervention will be assessed using the *Difficulties in Emotion Regulation Scale* (DERS; Gratz and Roemer, 2004). The DERS consists of 36 items answered on a 5-point scale and has been validated as a reliable measure (subscales: Cronbach's α ≥ .80; overall α =.93).
• Use of emotion regulation strategies: The *Heidelberg Form for Emotion Regulation Strategies* (HFERST; Izadpanah et al., 2019) assesses the frequency of emotion regulation strategy use for eight strategies (*rumination, reappraisal, acceptance, problem solving, suppression of emotional expression, suppression of emotional experience, avoidance, social support*). The questionnaire's 28 items are answered on 5-point scales, and the internal consistencies for the subscales range from α = .78 to α = .86 (Izadpanah et al., 2019).
• Ecological momentary assessment (EMA): Smartphone-based EMA will be implemented to capture eating disorder symptoms, everyday affect, and emotion regulation with a high ecological validity in daily life. EMA will be conducted for five days at pre- and post-treatment and will consist of five daily signal-contingent and additional event-based assessments after bulimic behaviors. Binge eating episodes and compensatory behaviors will be assessed based on the DSM-5 criteria for BN and previous EMA studies (APA, 2013; Munsch et al., 2009; Schaefer et al., 2020). In line with previous research, we will further measure BN symptoms, including shape and weight concerns (Hilbert et al., 2007) as well as eating-disorder urges (Tasca et al., 2009). The items measuring affect (Watson and Clark, 1994), emotion regulation strategies (Izadpanah et al., 2019), and regulatory difficulties (Lavender et al., 2017), were derived from previous research and established questionnaires (Pruessner et al., 2022).

#### 2.4.7 Other measures

• Attitudes towards psychological online interventions: Two subscales of the *Attitudes Towards Psychological Online Interventions Scale* (APOI; Schröder et al., 2015) will be used. The selected subscales consist of eight items rated on a 5-point scale and are validated as reliable measures of perceived technologization threat (Cronbach's α = .64) and anonymity benefits (Cronbach's α = .62) of online interventions (Schröder et al., 2015).
• Patient outcome expectancies: Treatment motivation will be assessed using the *Patients' Therapy Expectation and Evaluation Scale* (PATHEV; Schulte, 2008). The PATHEV consists of 16 items answered on a 5-point scale and has been revealed to reliably measure treatment motivation (α > .73; Schulte, 2005).
• Negative intervention effects: The *Negative Effects Questionnaire* (NEQ; Rozental et al., 2019) will be used to capture the occurrence of adverse intervention effects. For each of the 32 items of the NEQ, participants answer whether an adverse effect occurred (yes/no), how strong the effect was (0 to 4) and whether they attribute the negative effect on the treatment or something else. Two scores can be obtained, one for the frequency of adverse effects due to treatment, ranging from 0 to 32, and one for the negative impact, ranging from 0 to 128. The NEQ has been established as a valid and reliable measure and has an internal consistency of Cronbach's α = .95 (Rozental et al., 2019).
• Use of other healthcare services: To measure access to healthcare as well as the use of other healthcare services, the *Client Sociodemographic Service Receipt Inventory – European Version* (CSSRI-EU; original: Chisholm et al., 2000; Roick et al., 2001) will be used, which is a valid and reliable measure to assess the number and length of different types of healthcare service contacts (e.g., number of therapy sessions and contact with psychotherapists and psychiatrists).
• Patient adherence: The log files on the *Selfapy* platform will be employed to capture patient adherence. This includes the number of times and dates a participant logs into the web-based intervention and the number of completed modules. Additionally, participants in the IG will be asked how many times they used the intervention in the study assessments after six and 12 weeks.

### 2.5 Statistical methods

Our statistical analyses will consist of four steps: (1) descriptive analyses, (2) confirmatory analyses of our primary outcomes, including sensitivity analyses, (3) analyses of secondary outcomes, including sensitivity analyses, (4) moderator and mediator as well as analyses of the EMA data. All statistical analyses will be conducted using R Statistics (R Core Team, 2021); see the R script in the supplementary materials (Supplement S3).

#### 2.5.1 Primary and secondary outcome analyses

For the statistical analyses of the effectiveness of the web-based intervention for patients with BN, growth models within a mixed linear models (MLM) framework will be performed. These have the advantage of considering the multi-level data structure of three repeated assessments (level 1) as nested within patients (level 2) and treating the outcome change as a continuous process. Additionally, MLMs have more power when handling missing data than traditional approaches (Hesser, 2015; Kahn and Schneider, 2013; Tasca and Gallop, 2009). To evaluate whether there is a significant treatment × time interaction effect, we will follow a stepwise procedure by running models of increasing complexity (Kahn and Schneider, 2013). First, a random intercept model without predictors will be conducted, then we will add fixed effects of time (study entrance, 6 weeks, 12 weeks) and treatment (IG vs. CG) into the second model. Finally, the treatment × time interaction will be included to evaluate whether the outcome change differs between the IG and the CG. Likelihood ratio tests and the Akaike Information Criterion will be used to compare the fit of these three models. A significant treatment × time-interaction with more substantial changes in the intervention group compared to the waitlist group will confirm the hypotheses (Raudenbush and Bryk, 2002). The magnitude of treatment effects will be estimated following the suggestions for Cohen's *d* by Feingold, 2009, Feingold, 2013.

#### 2.5.2 Missing data and sensitivity analyses

Both completer analyses and intent-to-treat analyses will be conducted, as previous studies on web-based interventions report notable dropout rates (Linardon et al., 2018), and completers might systematically differ from non-completers (Altman et al., 2001). For this purpose, two sensitivity analyses will be run: (1) the conservative last-observation-carried-forward (LOCF) method, employing the last available measurement point of each subject, and (2) the multiple imputations by chained equations (MICE) approach using each participant's Body Mass Index, global eating psychopathology, number of binge-eating episodes determined in the EDE interview, and years since illness onset as predictors.

#### 2.5.3 Additional analyses

For a more detailed evaluation, additional statistical tests will include the analyses of potential moderators or confounding variables. Thus, we will check for possible pre-treatment differences between IG and CG in healthcare service utilization, demographic variables, and eating disorder symptom severity by conducting independent *t*-tests and chi-square tests. In case of significant group differences, these variables will be added as moderators of the treatment × time interaction effects to test the robustness of the findings. Further covariates possibly affecting the primary outcomes such as patient outcome expectancies, patient adherence, satisfaction, attitudes towards psychological online interventions, or emotion regulation will be included in our analyses.

Moreover, we will analyze the weekly assessments of eating disorder symptoms and the data from the EMA. This will allow us to examine changes in core eating disorder symptoms with a higher ecological validity and temporal resolution. As patient safety indicators, the percentage of participants in the IG who experienced adverse intervention effects caused by the intervention will be quantified, and the amount of impairment due to these adverse effects will be calculated. Further, changes in emotion regulation difficulties and strategy use in patients both on a habitual level and in daily life will be evaluated as potential moderators and mediators of symptom reduction in BN.

### 2.6 Statistical power and sample size

Sample size estimations were based on meta-analytic evidence for the effectiveness of psychological interventions for BN (Beintner et al., 2014; Haderlein, 2022; Svaldi et al., 2019). Due to the high heterogeneity of effect sizes ranging from medium to large (Beintner et al., 2014; Haderlein, 2022; Svaldi et al., 2019), the sample size needed to detect at least an effect of medium magnitude was estimated using the R package *powerlmm* (Magnusson, 2018). A power analysis with an effect size of *d* = 0.50, an intraclass-correlation of .40 (Arend and Schäfer, 2019), a power of .80, and an alpha-level of .05 resulted in a required sample of *N* = 152. Further power analyses, including possible dropouts, different effect sizes, and intra-class correlations, can be found in the supplementary materials (Figure S2), strengthening our conclusion that our sample size will be sufficiently large to detect a medium effect under different assumptions.

## 3 Discussion

Despite the effectiveness of psychotherapy for BN, only a limited number of patients with BN in routine care receive adequate evidence-based treatments (Kessler et al., 2013; Mack et al., 2014). This treatment gap, combined with the high illness-related burden and societal costs of BN, stresses the need to implement alternative treatments for BN (Bode et al., 2017; Kazdin et al., 2017). Applying web-based interventions in routine care might reduce barriers to traditional face-to-face therapies while providing more immediate low-threshold care for patients with BN (Aardoom et al., 2013; Aardoom et al., 2016; Beintner et al., 2014; Dölemeyer et al., 2013; Haderlein, 2022). However, more research on the effectiveness of web-based interventions for eating disorders is needed to evaluate their benefits for the health care system (Beintner et al., 2014; Haderlein, 2022; Linardon et al., 2020; Pittock et al., 2018; Taylor et al., 2021).

Our study aims to address this research gap by examining the effects of using a web-based self-help program for BN in routine care settings. Considering previous promising results concerning the effectiveness of such interventions for BN (Beintner et al., 2014; Haderlein, 2022; Pittock et al., 2018; Taylor et al., 2021), we aim to add knowledge to whether web-based interventions can reduce eating disorder symptoms and improve quality of life in routine care. Moreover, our goal is to explore changes in eating-disorder-related topics such as self-esteem and emotion regulation to better understand possible mechanisms of change.

### 3.1 Strengths and challenges

Based on our goal to evaluate the effectiveness of a web-based intervention, our study was designed as a randomized controlled trial with a waitlist control group maximizing the internal validity of results. To further decrease systematic biases, our randomization will follow the principles of sequence allocation concealment, and systematic pre-treatment differences will be minimized by excluding individuals receiving psychotherapy or pharmacotherapy for BN at baseline (Altman et al., 2001; Gupta, 2011). Further, implementing the web-based intervention into a routine care setting will increase the study's external validity. As such, we enhance the generalizability of our results by allowing participants in our trial to receive care as usual after randomization in both groups. Additionally, participants meeting the criteria for other mental disorders will not be excluded as long as their psychological condition allows them to securely participate in a web-based intervention.

Moreover, we aim at contributing to the research on treatments for BN by investigating changes in both cognitive and behavioral eating disorder symptoms. Network analyses indicate that it is possible to differentiate between various types of BN symptoms (Forrest et al., 2018; Levinson et al., 2017), including the number of binge eating episodes, compensatory behaviors, and global eating pathology. Therefore, we will examine changes in these facets separately to better understand whether and how web-based interventions influence different types of BN symptoms. Weekly assessments and naturalistic EMA data will further increase the ecological validity of results and provide more detailed insights into trajectories of change while reducing retrospective biases (Munsch et al., 2007; Munsch et al., 2019).

Apart from investigating changes in eating disorder symptoms, we will analyze whether and how the web-based intervention can help improve participants' daily life. Eating disorders are associated with a broad range of adverse outcomes, and thus examining changes in psychosocial functioning, well-being, and work capacity will help evaluate the web-based intervention from a broader perspective (Colmsee et al., 2021; De Jong et al., 2013; Levinson et al., 2017; Svaldi et al., 2012). Finally, our study will be one of the first to investigate possible mechanisms of change, such as alterations in self-esteem and emotion regulation, which might help advance BN treatment in the future.

Nevertheless, these strengths will probably be associated with some limitations. First of all, to conduct our analyses with adequate power, a high number of participants needs to be included in our study. The recruitment of such a large-scaled sample might be challenging because the prevalence of BN is relatively low, and barriers in the help-seeking process might limit the number of eligible and interested patients (Ali et al., 2016; Becker et al., 2010; Grange and Loeb, 2007; Kessler et al., 2013; Mond, 2014; Puhl and Suh, 2015). To maximize the number of participants and reach the intended sample size, we will employ various recruitment methods, including online and offline advertisement and allowing location-independent participation.

Additionally, the current study does not employ follow-up assessments to investigate the long-term effectiveness of the web-based BN intervention. In general, BN is associated with a high risk for relapses, stressing the need for data on long-term symptom courses following treatment (Quadflieg and Fichter, 2019; Stice et al., 2009). However, follow-up assessments increase the burden for participants and result in longer waiting periods for the control group. Since the web-based program for BN was developed as an add-on to routine care and not as a replacement for face-to-face therapy, the current trial primarily investigates whether adding it into standard clinical practice can immediately improve patients' mental health and quality of life. Moreover, there is no empirical evidence for the effectiveness of the present BN intervention, so focusing on the short-term benefits seems reasonable before testing long-term effects. Still, future studies, including follow-up assessments, should be conducted if the effectiveness of the web-based program can be confirmed.

Another challenge that might occur is a high dropout from the study, which has been frequently reported in previous trials on interventions for eating disorders (Linardon et al., 2018). Low adherence might be especially problematic for the present study, as the high number of assessments combining traditional questionnaires with EMA will lead to a relatively high burden for participants. However, the financial reimbursement and a reminder system, including text messages, emails, and phone calls, will be implemented to encourage participants and hopefully increase adherence (Treweek et al., 2013). Further, we limited the EMA period to five days and included rather short assessment scales respectively only included subscales when possible. Concerning the data analysis, we will follow the intention-to-treat approach and conduct sensitivity analyses to be able to evaluate the influence of possible dropouts (Gupta, 2011). In sum, we are optimistic about being able to assess a high number of outcomes to be able to address our research goals and increase the external validity of results by adding EMA, while keeping dropout rates low.

### 3.2 Conclusion

In sum, the contributions of the current study to the growing field of web-based interventions for BN are threefold. First of all, our trial is designed to evaluate whether applying web-based self-help interventions in routine care of BN can help reduce eating disorder symptoms, improve the overall quality of life, and illness-related topics such as psychosocial functioning. Secondly, our study will add knowledge on potential predictors of treatment outcomes and mechanisms of change. This will help us better understand how web-based self-interventions may reduce the burden for patients with BN. Third, by including a broad range of assessment methods and different outcomes instead of focusing only on changes in eating disorder symptoms, we will be able to further investigate whether and how web-based interventions impact the daily life of participants. Based on these innovations, the long-term goal of our research on web-based interventions is to implement evidence-based treatments into routine care, reduce social costs, and enhance the quality of life for individuals with eating disorders.

## Abbreviations

APA: American Psychiatric Association
APOI: Attitudes Towards Online Interventions
AZ: Aktenzeichen [file number]
BMI: Body Mass Index
BN: bulimia nervosa
BPtK: Bundespsychotherapeutenkammer [German national association of psychotherapists]
CBT: Cognitive-Behavioral Therapy
CG: control group
CIA: Clinical Impairment Assessment
CONSORT: Consolidated Standards of Reporting Trials
CSSRI: Client Sociodemographic Service Receipt Inventory
DERS: Difficulties in Emotion Regulation Scale
DSM: Diagnostic and Statistical Manual of Mental Disorders
DIPS-OA: Diagnostic Interview for Psychological Disorders-Open Access
EDE: Eating Disorder Examination Interview
EDE-Q: Eating Disorder Examination Questionnaire
EMA: ecological momentary assessment
EU: European Union
GAD-7: Generalized Anxiety Disorder Scale
HFERST: Heidelberg Form for Emotion Regulation Strategies
ICC: intraclass correlation coefficient
IG: intervention group
*i*PCQ: Productivity Cost Questionnaire
LOCF: last observation carried forward
MICE: multiple imputations by chained equations
MLM: mixed linear modeling
*i*MTA: Institute for Medical Technology Assessment
NEQ: Negative Effects Questionnaire
PATHEV: Patients' Therapy Expectation and Evaluation Scale
PHQ: Patient Health Questionnaire
PICO: Population Intervention Compared Outcome
RSES: Rosenberg Self-Esteem Scale
SPIRIT: Standard Protocol Items: Recommendations for Interventional Trials
WBQ: Weekly Binges Questionnaire
WHO-5: World Health Organization-5 Well-Being Index

## Trial status

Recruitment started in January 2021 and is still ongoing. The first patient was enrolled in the study on January 25th, 2021. Assessments are expected to be completed by May 2022.

## Funding

The study will be funded by a 10.13039/501100008530European Regional Development Fund awarded to *Selfapy*. Publication fees will be financially supported by the *German Research Foundation* (Deutsche Forschungsgemeinschaft) Open Access Publishing Fund at Heidelberg University. The funders have no authority over the study design, collection, management, analysis, and interpretation of data, writing of the report, and the decision to submit the findings for publication.

## Ethics approval and consent to participate

Ethics approval has been obtained from the institutional review board at Heidelberg University (AZ Tim 2020 1/1). Informed consent will be obtained from all participants, and the trial will be conducted in compliance with the Declaration of Helsinki and good clinical practice. International data privacy regulations and EU legislation will be considered.

## Availability of data and material

The de-identified and anonymized data and the R analysis script of the current trial will be made available on the *Open Science Framework* (https://osf.io/zdnea/?view_only=b204c63d3d6c4d569e5a140927708eb6).

## CRediT authorship contribution statement

LP and CT designed the study. SH and LP performed the sample size calculations and drafted the statistical design of the trial. SH and LP wrote the first draft of the manuscript. All co-authors (LP, JR, CL, SB, CT) contributed to critical revisions of the paper and approved the final manuscript.

## Declaration of competing interest

The authors declare that they have no known competing financial interests or personal relationships that could have appeared to influence the work reported in this paper.

## Appendix A Supplementary material

Supplementary material Image 1
