# Supplementary material for: Applying a web-based self-help intervention for bulimia nervosa in routine care: Study protocol for a randomized controlled trial
Source: Internet Interv. 2022 Feb 17;28:100512. doi: 10.1016/j.invent.2022.100512 (PMC8894237; doi:10.1016/j.invent.2022.100512)
Supplement: Supplementary file 1 — Supplementary material [file mmc1.docx]

Supplementary Material

for

**Applying a web-based self-help intervention for bulimia nervosa in routine care: Study protocol for a randomized controlled trial**

Hartmann, Steffen^1^;

Pruessner, Luise^1^;

Rubel, Julian A.^2^;

Lalk, Christopher^2^;

Barnow, Sven^1^;

Timm, Christina^1^

^1^ Department of Psychology, Heidelberg University, Hauptstr. 47-51, 69117 Heidelberg

^2^ Psychotherapy Research Unit, University of Giessen, Otto-Behaghel-Straße 10, 35394 Giessen

**PICO Study Overview**

Table S1

Overview of the study based on the PICO framework.

| Criteria | Assessments |
| --- | --- |
| **P:**  Population | *Inclusion criteria:*   1. age between 18 and 65 years 2. sufficient German language skills (C1) 3. a smartphone with permanent internet access during the study period 4. meeting the diagnostic criteria for bulimia nervosa according to the Diagnostic- and Statistical Manual of Mental Disorders (DSM-5)   *Exclusion criteria:*   1. current severe depressive episode 2. acute suicidality 3. comorbid bipolar disorder or psychotic disorder 4. acute substance dependence 5. current psychotherapy or pharmacotherapy for eating disorders 6. Body Mass Index (BMI) below 18.5 |
| **I:**  Intervention | - 12 weeks of a web-based intervention for bulimia nervosa (*Selfapy)* |
| **C:**  Control | - 12 weeks of waiting time |
| **O:**  Outcome | *Primary Confirmatory*   - Number of binge eating episodes and compensatory behaviors (EDE-Q)   *Secondary Confirmatory*   - Global eating psychopathology (EDE-Q) - Weekly Binges Questionnaire (WBQ) - Clinical impairment (CIA) - Well-being (WHO-5) - Work capacity (*i*PCQ)   *Secondary Exploratory*   - Comorbid depression (PHQ-9) - Comorbid anxiety (GAD-7) - Self-esteem (RSES) - Difficulties in emotion regulation (DERS) - Emotion regulation frequencies (HFERST) - Everyday eating disorder symptoms and emotion regulation (EMA) |

**
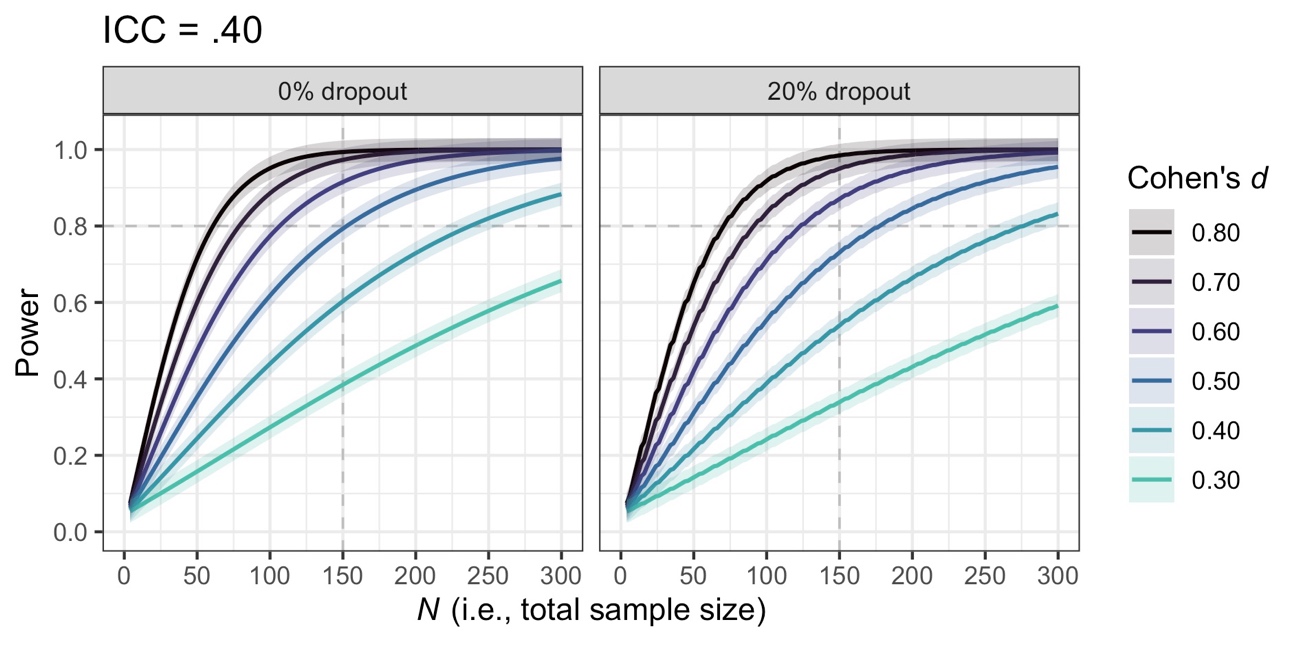
Power Analyses**

**
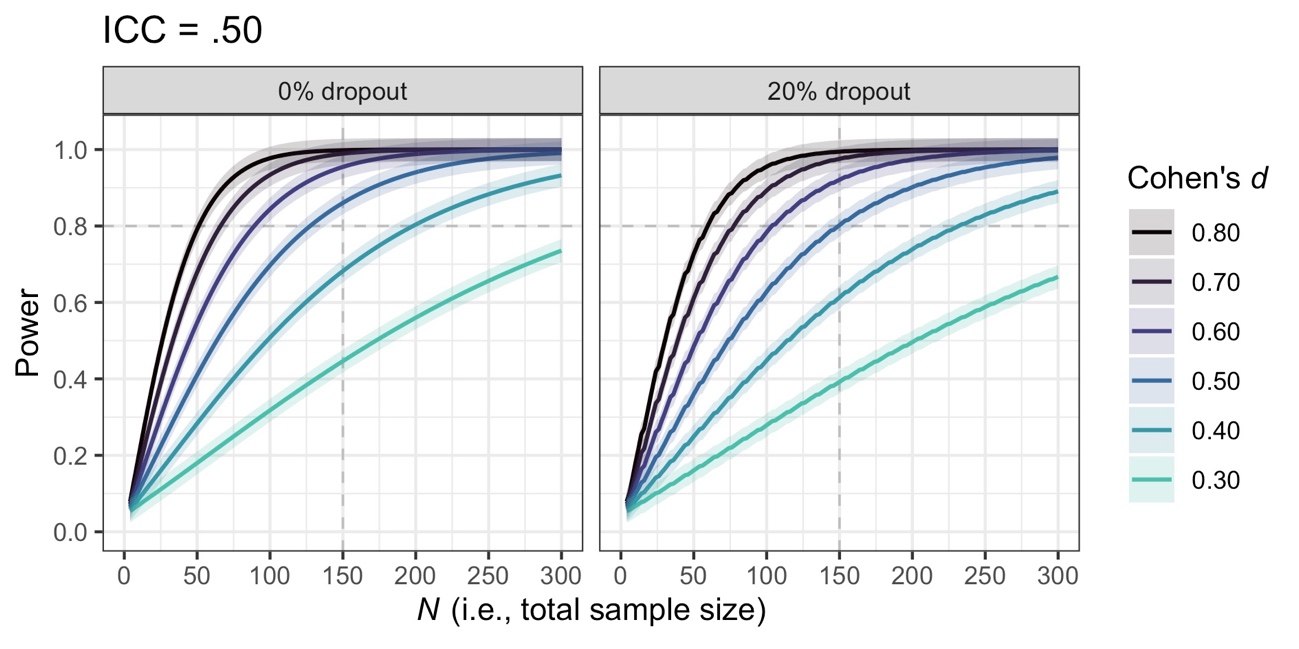
**

**
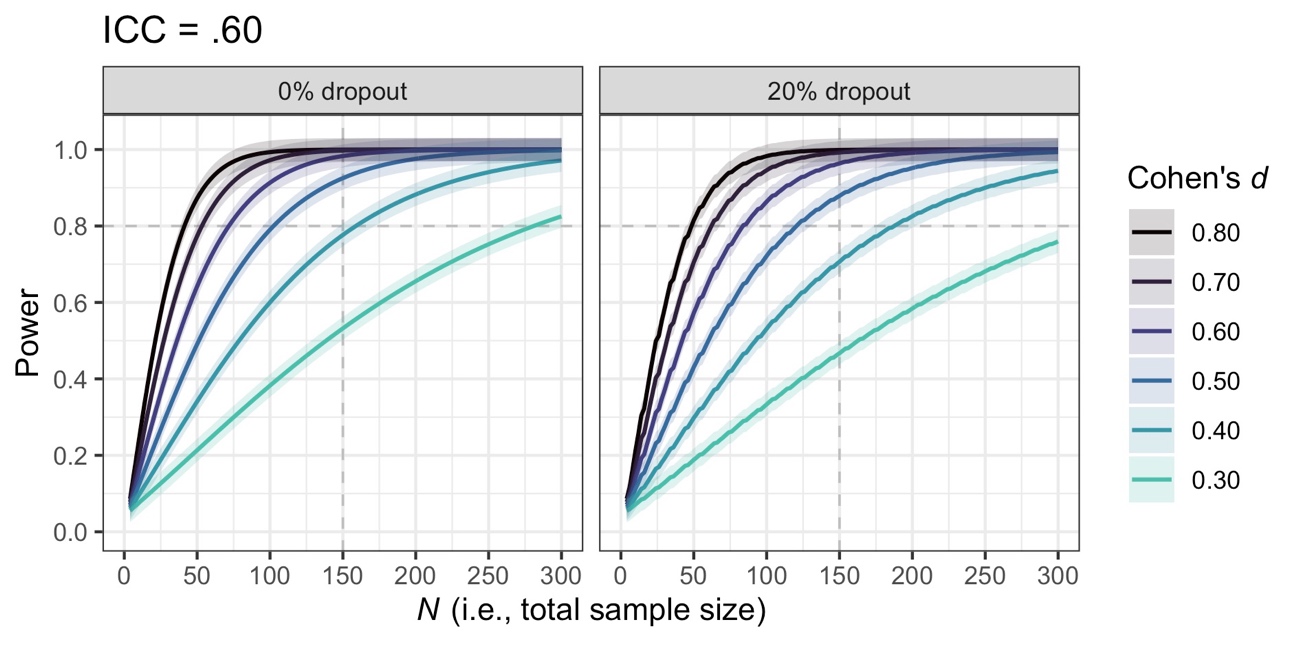
**

*Figure S2*. Power analyses are based on the following parameters: α = .05, power = .80, number of groups = 2, number of measurements = 3.

**R Script of the Confirmatory Analyses**

Supplement S3

*Script for the confirmatory data analyses*

**#COMPLETER- analysis---**

*#reshape dataset:*

df_long = reshape(data = df,

idvar = "ID",

direction = "long",

varying = list(c("Y1","Y2","Y3")),

timevar = "TIME",

times = paste(0:2),

v.names = "Y")

df_long$TIME = as.numeric(df_long$TIME)

*#calculation:*

mod0 <- lmer(Y ~ 1 + (1|ID), data= df_long)

mod1 <- lmer(Y ~ TIME + (1|ID), data= df_long)

mod2 <- lmer(Y ~ TIME*GROUP + (1|ID), data= df_long)

anova(mod0, mod1, mod2)

**#LOCF imputation ----**

LOCF_df = df

LOCF_df$Y2[is.na(LOCF_df$Y2)] <- LOCF_df$Y1[is.na(LOCF_df$Y2)]

LOCF_df$Y3[is.na(LOCF_df$Y3)] <- LOCF_df$Y2[is.na(LOCF_df$Y3)]

*#reshape dataset:*

LOCF_df_long = reshape(data = LOCF_df,

idvar = "ID",

direction = "long",

varying = c("Y1", "Y2","Y3"),

timevar = "TIME",

times = paste(0:2),

v.names = "Y")

LOCF_df_long$TIME = as.numeric(LOCF_df_long$TIME)

*#calculation:*

mod0.locf <- lmer(Y ~ 1 + (1|ID), data= LOCF_df_long)

mod1.locf <- lmer(Y ~ TIME + (1|ID), data= LOCF_df_long)

mod2.locf <- lmer(Y ~ TIME*GROUP + (1|ID), data= LOCF_df_long)

anova(mod0.locf, mod1.locf, mod2.locf)

**#MICE imputation**

*# imputation based on control group data:*

ignore_vector <- df$GROUP

ignore_vector <- as.logical(ignore_vector)

*#MICE imputation, 5 imputations, ignore intervention group:*

imp_mice <- mice(data = df, m = 5, ignore = ignore_vector)

*#transform mice object into R list:*

df_mice <- as.list(1:5)

for(i in 1:5){

df_mice[[i]] <- mice::complete(imp_mice, action=i) }

*#transform to long format:*

df_mice_long <- lapply(df_mice, pivot_longer, cols = c("Y1","Y2","Y3"), names_to = "times", values_to = "Y")

i = 1

while (i < 6)

{

df_mice_long[[i]]$TIME <- 0

df_mice_long[[i]]$TIME[df_mice_long[[i]]$times == "Y2"] <- 1

df_mice_long[[i]]$TIME[df_mice_long[[i]]$times == "Y3"] <- 2

df_mice_long[[i]]$TIME = as.numeric(df_mice_long[[i]]$TIME)

i = i + 1

}

*#calculation:*

mod0.mice <- lmer(Y ~ 1 + (1|ID), data= df_mice_long)

mod1.mice <- lmer(Y ~ TIME + (1|ID), data= df_mice _long)

mod2.mice <- lmer(Y ~ TIME*GROUP + (1|ID), data= df_mice _long)

anova(mod0.mice, mod1.mice, mod2.mice)

**#calculation of effect size**

*#extract effect:*

b3 = as.numeric(fixef(model)['TIME:GROUP'])

*#calculation of pooledSD:*

SD_raw_pre_t <- filter(df, GROUP == "1") %>% summarise(s = sd(Y1)) %>% pull() # treatment baseline SD

SD_raw_pre_c <- filter(df, GROUP == "0") %>% summarise(s = sd(Y1)) %>% pull() # control baseline SD

*#insert group size:*

n_t <- 76

n_c <- 76

SD = sqrt(((n_t - 1) * SD_raw_pre_c^2 + (n_c - 1) * SD_raw_pre_t^2) / (n_t + n_c - 2))

*#Cohen's d:*

time = 2

d = b3*2/SD

*#CI:*

b_SE = summary(model)$coef[4, 2, drop = FALSE][1,1]

LCL_b = b3 - (1.96*b_SE)

UCL_b = b3 + (1.96*b_SE)

LCL_d = (LCL_b*2)/SD

UCL_d = (UCL_b*2)/SD
